# Necrosis drives susceptibility to *Mycobacterium tuberculosis* in Polg^D257A^ mutator mice

**DOI:** 10.1128/iai.00324-24

**Published:** 2025-02-19

**Authors:** C. J. Mabry, C. G. Weindel, L. W. Stranahan, J. J. VanPortfliet, J. R. Davis, E. L. Martinez, A. P. West, K. L. Patrick, R. O. Watson

**Affiliations:** 1 Department of Microbial Pathogenesis and Immunology, Texas A&M Health, College of Medicine 550026 , Bryan, Texas, USA; 2 Department of Veterinary Pathobiology, Texas A&M College of Veterinary Medicine and Biomedical Sciences 199062 , College Station, Texas, USA; 3 The Jackson Laboratory 1514 , Bar Harbor, Maine, USA; 4 Department of Medicine, Division of Infectious Diseases, Vanderbilt University Medical Center 12328 , Nashville, Tennessee, USA; 5 Department of Pathology, Microbiology, and Immunology, Vanderbilt University Medical Center 204907 , Nashville, Tennessee, USA; Rutgers-New Jersey Medical School, Newark, New Jersey, USA

**Keywords:** Polg, *Mycobacterium tuberculosis*, innate immunity, necrosis

## Abstract

The genetic and molecular determinants that underlie the heterogeneity of *Mycobacterium tuberculosis* (Mtb) infection outcomes in humans are poorly understood. Multiple lines of evidence demonstrate that mitochondrial dysfunction can exacerbate mycobacterial disease severity, and mutations in some mitochondrial genes confer susceptibility to mycobacterial infection in humans. Here, we report that mutations in mitochondria DNA (mtDNA) polymerase gamma potentiate susceptibility to Mtb infection in mice. Polg^D257A^ mutator mtDNA mice fail to mount a protective innate immune response at an early infection time point, evidenced by high bacterial burdens, reduced M1 macrophages, and excessive neutrophil infiltration in the lungs. Immunohistochemistry reveals signs of enhanced necrosis in the lungs of Mtb-infected Polg^D257A^ mice, and Polg^D257A^ mutator macrophages are hypersusceptible to extrinsic triggers of necroptosis *ex vivo*. By assigning a role for mtDNA mutations in driving necrosis during Mtb infection, this work further highlights the requirement for mitochondrial homeostasis in mounting balanced immune responses to Mtb.

## INTRODUCTION

Defects in mitochondria are associated with a variety of human diseases and metabolic disorders (1). Inherited mitochondrial diseases, caused by mutations harbored in the mitochondrial genome (mtDNA) or in nuclear-encoded mitochondrial genes, lead to devastating neurodegenerative and cardiovascular disorders (2). They are also frequently associated with chronic inflammation and autoimmunity. Type I interferonopathies, for example, are often caused by mutations that result in aberrant release of mitochondrial nucleic acids and chronic engagement of cytosolic nucleic acid sensing (3–5). Consistent with important links between mitochondrial health and immune function, patients with mitochondrial mutations are prone to frequent viral and bacterial infections that are often severe (6, 7). They can also suffer from systemic inflammatory response syndrome caused by hyperinflammation in the absence of infection (8). Furthermore, human single nucleotide polymorphisms (SNPs) in mitochondrial genes have been shown to confer susceptibility to several bacterial pathogens, including *Salmonella enterica* serovar Typhi (9), *Listeria monocytogenes* (10), and *Mycobacterium leprae* (11). Despite clear connections between mitochondria and immunity, the mechanistic contributions of specific mitochondrial-associated mutations in driving susceptibility to infection remain unclear.

*Mycobacterium tuberculosis* (Mtb) is the causative agent of tuberculosis, an ancient disease that remains the number one infectious disease killer worldwide (12). The success of Mtb is driven in large part by its ability to manipulate and establish a replicative niche in host innate immune cells, primarily macrophages (13). Mitochondria serve as a key hub in the Mtb-macrophage host-pathogen interface, as they control relevant processes such as cellular energetics, lipid metabolism, and reactive oxygen species (ROS) generation. Crucially, mitochondria also regulate commitment to programmed cell death pathways (14). While the precise molecular determinants of TB disease severity remain elusive, decades of research have identified a role for cell death in dictating TB disease outcomes. Apoptosis or “immunologically silent” cell death correlates with protection, whereas necrotic forms of cell death are associated with mycobacterial cell-to-cell spread and immunopathology (15–18). Consequently, TB has evolved a variety of strategies to inhibit apoptosis while promoting necrosis (19).

Several studies have shown how distinct modes of cell death (e.g., inflammasome-mediated pyroptosis, necroptosis, and ferroptosis) promote Mtb pathogenesis (20–24). We and others have begun to link cell death during Mtb infection with disruptions in mitochondrial homeostasis (14, 25, 26). We recently reported that mutations in the mitochondrial-associated protein leucine-rich repeat kinase 2 (LRRK2) can promote necroptosis during Mtb infection by rendering mitochondrial membranes susceptible to damage by the pore-forming protein gasdermin D, resulting in the release of mitochondrial ROS (26). Building on this finding, we set out to determine how other mitochondrial mutations alter Mtb infection outcomes.

Here, we investigate how Mtb infection proceeds in a common model of mitochondrial dysfunction, the Polg mutator mouse. These mice contain a knock-in D257A mutation in the exonuclease domain of Polg, the sole mitochondrial DNA polymerase (27). This mutation disrupts the proofreading ability of Polg and subsequently results in the accumulation of mtDNA mutations in mice as they age (27). Although this mouse model does not harbor a specific human disease-associated mutation, it presents with symptoms that parallel many of the clinical manifestations seen in mitochondrial disease patients, such as cardiomyopathy, progressive hearing loss, and anemia (28). These mice also have exacerbated inflammatory responses and are susceptible to septic shock (3). They have been useful tools for studying mitochondrial dysfunction in aging (27, 29), but only a few studies have used them to study infection (30). Here, we report that Polg^D257A^ mutator mice display enhanced susceptibility to Mtb infection evidenced by higher bacterial burdens, excessive neutrophil infiltration in the lung, and enhanced necrosis. These findings argue that mtDNA fidelity is required to maintain balanced cell death outcomes during Mtb infection.

## RESULTS

### Polg^D257A^ mutator mice harbor higher Mtb bacterial burdens

**Fig 1:**
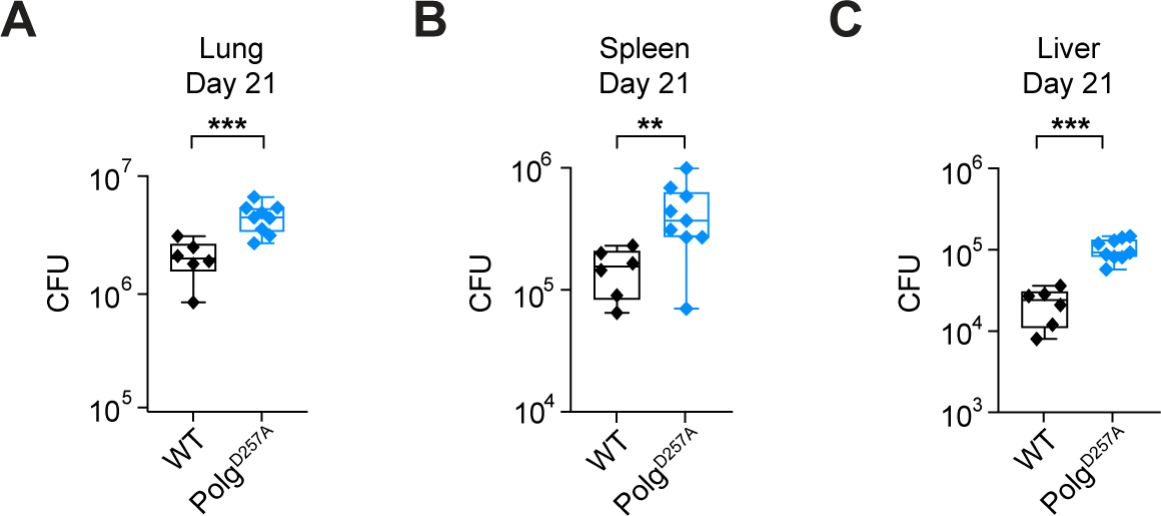
Polg^D257A^ mutator mice display increased Mtb replication and dissemination *in vivo*. Mtb CFUs collected from WT and Polg^D257A^ mice lungs (**A**), spleens (**B**), and livers (**C**) at day 21 post-Mtb infection. Statistical analysis: *P < 0.05, **P < 0.01, ***P < 0.001, and ****P < 0.0001. Statistical significance was determined for panels **A–C** using Mann-Whitney *U* test.

To better understand how mitochondrial-associated gene mutations drive Mtb infection outcomes, we chose to study the Polg^D257A^ mutator mouse model (B6.129S7(Cg)-*Polg*^*tm1Prol*^/J). These mice accumulate linear mtDNAs and fragmented mtDNA genomes that contribute to mitochondrial dysfunction and aging (27). Using a low-dose aerosol model, which administers 100–200 Mtb bacilli into the lungs of each animal, we infected WT (*n* = 6) and Polg^D257A^ mutator (*n* = 9) mice at approximately 3 months of age. We chose to infect young mice to avoid variability in mtDNA instability and the potential for inflammation-associated pathology in our cohort (Polg^D257A^ mutator mice experience premature aging and early death [27–29]). At 21 days post-infection, we sacrificed mice, homogenized a portion of the lung, spleen, and liver, and enumerated Mtb colony-forming units (CFUs) to measure bacterial burden. We observed a significant increase (half-log) in Mtb recovered from the lungs of Polg^D257A^ mutator mice compared to WT mice (Fig. 1A). Polg^D257A^ mutator mice were also more permissive to Mtb dissemination, as bacterial burdens were higher in their spleens and livers (Fig. 1B and C). These findings demonstrate that mice experiencing Polg-dependent mitochondrial dysfunction cannot properly control Mtb replication and spread *in vivo*.

### Polg^D257A^ mutator mice lungs exhibit increased neutrophils and fewer M1 macrophages during Mtb infection

**Fig 2:**
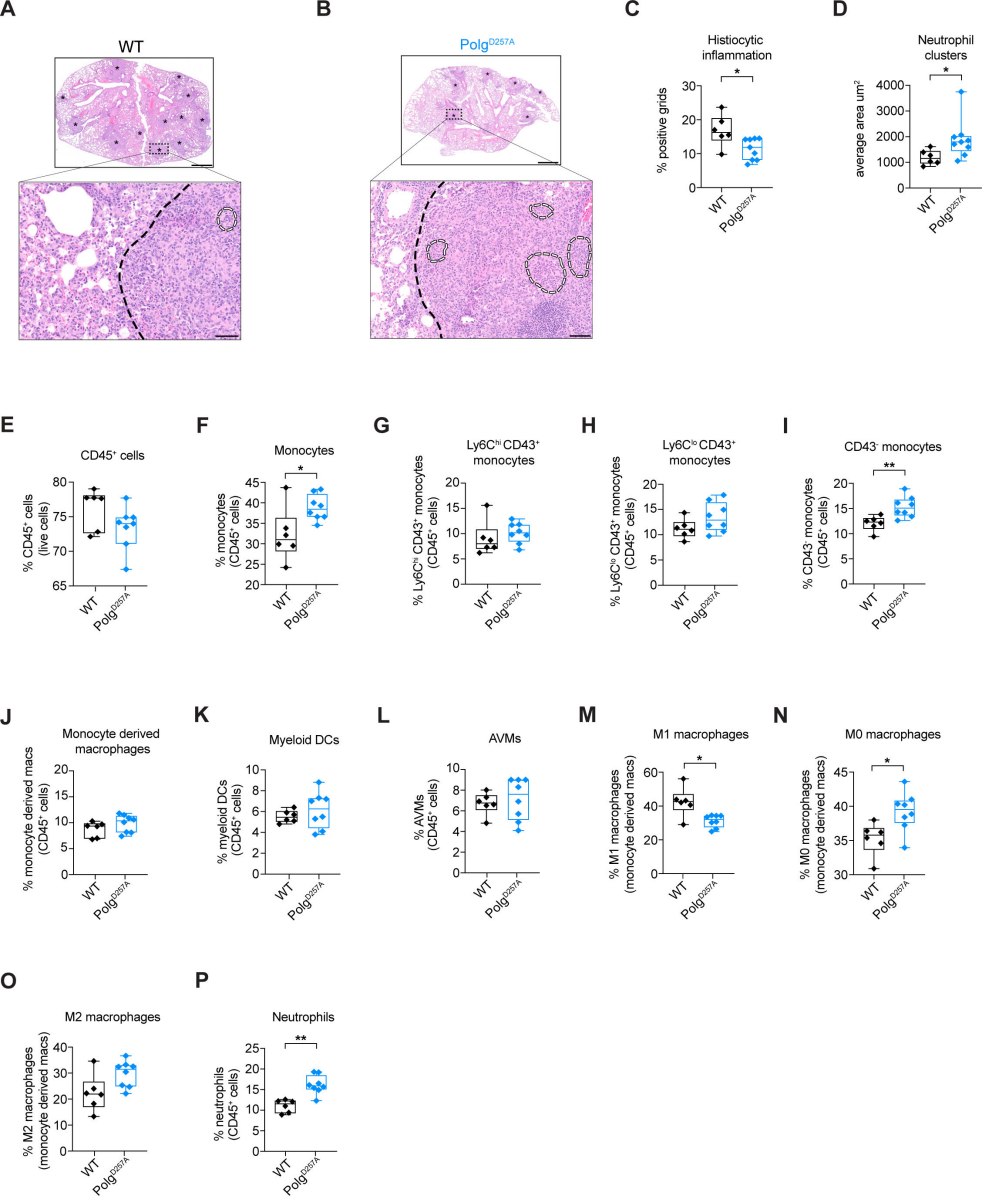
Polg^D257A^ mutator mice display increased neutrophils and less M1 macrophages during Mtb infection. Hematoxylin and eosin staining of WT (**A**) and Polg^D257A^ (B) mice lungs at day 21 post-Mtb infection. Asterisks denote areas consolidated and infiltrated by inflammatory cells. Black dashed line delineates regions of healthy versus inflamed areas of the lung in the lower panel. Outlined regions (white dashed lines) denote areas of neutrophil clusters. Upper panel scale bar is 800 µm. Lower panel scale bar is 20 µm. (**C**) Quantification of histiocytic inflammation using H&E staining of WT and Polg^D257A^ mice lungs at day 21 post-Mtb infection. Each data point represents the percentage of positive grids with histiocytic inflammation in WT and Polg^D257A^ mice Mtb-infected lungs. (**D**) Average total surface area of neutrophil clusters using H&E staining of WT and Polg^D257A^ mice lungs at day 21 post-Mtb infection. For flow cytometry analysis, WT (*n* = 6) and Polg^D257A^ (*n* = 8) mice lungs were analyzed. Lung CD45^+^ cells (**E**), monocytes (**F**), Ly6C^hi^ CD43^+^ monocytes (**G**), Ly6C^lo^ CD43^+^ monocytes (**H**), CD43^−^ monocytes (**I**), monocyte-derived macrophages (**J**), myeloid dendritic cells (**K**), alveolar macrophages (**L**), M1 macrophages (**M**), M0 macrophages (**N**), M2 macrophages (**O**), and neutrophils (**P**) in WT and Polg^D257A^ mice analyzed by flow cytometry at day 21 post-Mtb infection. Statistical analysis: *P < 0.05, **P < 0.01, ***P < 0.001, and ****P < 0.0001. Statistical significance was determined for panels **C–P** using Mann-Whitney *U* test.

To grossly examine the inflammatory state of the lungs in Mtb-infected Polg^D257A^ mutator mice, we evaluated lung tissues via hematoxylin and eosin (H&E) staining. As is characteristic of Mtb infection, we observed histiocytic inflammation (aggregation of monocyte- and macrophage-derived cells) in the lungs of both genotypes (Fig. 2A and B). Curiously, despite harboring higher bacterial loads, Polg^D257A^ mutator mice displayed less histiocytic lung inflammation at day 21 post-infection compared to WT (Fig. 2C, black outline in H&E images). This suggests that Polg^D257A^ mutator mice fail to effectively recruit one or more types of immune cells into infected regions of the lung. In contrast, we found that Polg^D257A^ mutator mice lungs were more prone to neutrophil cluster formation, a phenomenon typically associated with NET release and pathogen clearance (31, 32) (Fig. 2D, white outline in H&E images). These findings argue that an altered immune cell landscape dominates the lungs of Polg^D257A^ mutator mice during Mtb infection.

To further define innate immune cell populations in the lungs of Polg^D257A^ mutator mice during Mtb infection, we isolated single-cell suspensions and used flow cytometry to identify key cell types. Overall, we measured similar numbers of hematopoietic cells (CD45^+^) in the lungs of Mtb-infected Polg^D257A^ mutator and WT mice (Fig. 2E). However, the percentage of total monocytes, in particular, classical CD43^-^ monocytes, was higher in Polg^D257A^ mutator mice (Fig. 2F through I). Previous studies have correlated CD43 with control of Mtb in macrophages, and in mice, the absence of CD43 leads to increased bacterial load and defective granuloma formation (33). We did not observe major differences in the percentage of monocyte-derived macrophages (CD45^+^ CD11b^+^ Ly6C^-^ SSC^hi^) (Fig. 2J), dendritic cells (CD45^+^ CD11b^+^ CD11c^+^) (Fig. 2K), or alveolar macrophages (CD45^+^ CD11b^low/neg^ CD11c^+^ CD170^+^) (Fig. 2L).

Consistent with the lower levels of histiocytic inflammation (Fig. 2C), we measured fewer M1 macrophages (CD86^+^ macrophages) in Mtb-infected Polg^D257A^ mutator mice (Fig. 2M). This was concomitant with an increased percentage of M0 (CD86^−^ CD206^−^) macrophages (Fig. 2N), suggesting a macrophage polarization defect in Polg^D257A^ mutator mice. M2 (CD86^−^ CD206^+^) macrophages were similar between the two genotypes (Fig. 2O). Importantly, pro-inflammatory M1 macrophages play an important role in the early control of Mtb by promoting granuloma formation, triggering T cell infiltration, and upregulating antimicrobial molecules like nitric oxide (34–36). Concomitant with fewer M1 macrophages, we observed a significant increase in the recruitment of neutrophils (CD45^+^ C220^−^ CD11b^+^ Ly6G^+^) in Polg^D257A^ mice lungs (Fig. 2P). Enhanced neutrophil recruitment has been previously shown to correlate with tissue damage and poor Mtb infection outcomes in both animal models and tuberculosis patients (37–39). Collectively, these results suggest that a combination of defective M1 macrophage polarization, low expression of CD43 on monocytes, and/or enhanced neutrophil infiltration results in higher Mtb bacterial burdens in Polg^D257A^ mice.

### Decreased M1 macrophage responses drive susceptibility to Mtb infection in Polg^D257A^ mutator mice

Previous studies have linked Polg-related mitochondria dysfunction to elevated type I IFN expression following exposure to pattern recognition receptor agonists such as LPS and dsDNA (3). To begin to understand if such a defect contributes to Mtb susceptibility in Polg^D257A^ mutator mice, we isolated primary bone marrow-derived macrophages (BMDMs) from WT and Polg^D257A^ mice and infected them with Mtb (MOI = 5). We did not observe differences in the ability of Polg^D257A^ BMDMs to elicit *Ifnb1* or interferon-stimulated gene (ISG) transcript expression in response to Mtb (Fig. S2A), nor did we measure differences in the expression of VIPERIN (encoded by *Rsad2*) at the protein level 24 h post-Mtb infection (Fig. S2B). These data suggest that the cell-intrinsic ability of Polg^D257A^ macrophages to generate type I IFN in response to Mtb is intact *ex vivo*.

**Fig 3:**
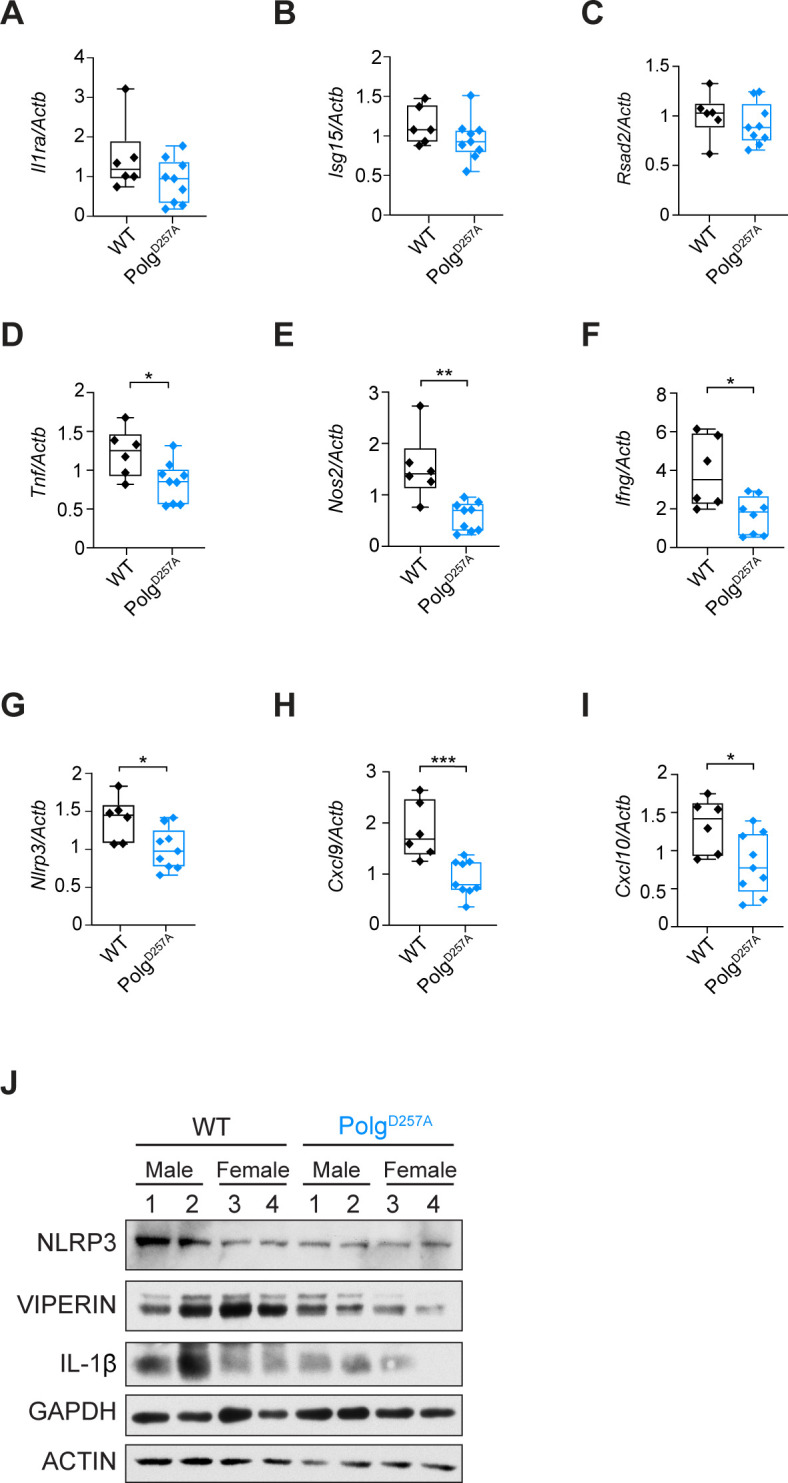
Dampened M1 macrophage pro-inflammatory response is a hallmark of Polg^D257A^ mutator mice during Mtb infection. Lung *Il1ra* (**A**), *Isg15* (**B**), *Rsad2* (**C**), *Tnf* (**D**), *Nos2* (**E**), *Ifng* (**F**) *Nlrp3* (**G**), *Cxcl9* (**H**), and *Cxcl10* (**I**) transcripts in WT and Polg^D257A^ mice analyzed by qRT-PCR at day 21 post-Mtb infection. Transcripts were normalized to *Actb* transcript levels. (**J**) Immunoblot analysis of NLRP3, VIPERIN, and IL-1β protein levels in WT and Polg^D257A^ mouse lungs at day 21 post-Mtb infection. GAPDH and ACTIN were used as loading controls. Statistical analysis: *P < 0.05, **P < 0.01, ***P < 0.001, and ****P < 0.0001. Statistical significance was determined for panels **A–I** using Mann-Whitney *U* test.

We next asked how cytokine expression was altered in Mtb-infected Polg^D257A^ mutator mice. In lung homogenates, ISGs *Il1ra*, *Isg15*, and *Rsad2* (which encodes VIPERIN) were expressed similarly in WT and Polg^D257A^ Mtb-infected lungs (Fig. 3A through C). However, consistent with low M1 macrophage numbers (Fig. 2M), pro-inflammatory transcripts like *Tnf*, *Nos2*, *Ifng*, *Nlrp3*, *Cxcl9*, and *Cxcl10* were less abundant in the Polg^D257A^ lungs (Fig. 3D through I). Other pro-inflammatory cytokines (*Il1b* and *Il6*) and chemokines (*Cxcl1 and Cxcl5*) were consistently expressed by both genotypes (Fig. S2C through F). Because transcript levels do not always correlate with protein levels, we also performed western blot analysis on lung homogenates. Although most secreted cytokines are undetectable in lung homogenates, we were able to confirm lower expression of NLRP3 at the protein level (Fig. 3J). Curiously, although *Rsad2* and *Il1b* transcript abundance was similar in WT and Polg^D257A^ lungs, protein levels were, on average, lower in Polg^D257A^ (Fig. 3J). Collectively, these data demonstrate that Polg^D257A^ mice generate a weak pro-inflammatory milieu in the lung during early Mtb infection.

Finally, looking beyond the lung, we measured circulating cytokines in Mtb-infected WT and Polg^D257A^ mice. Briefly, we used the ProcartaPlex Multiplex Immunoassay Kit to analyze serum isolated from WT (*n* = 6) and Polg^D257A^ (*n* = 9) mice. Most of the analytes in our 12-plex panel were below the level of detection. For the analytes we could measure (IFN-γ, TNF-α, CXCL5, CXCL9, and CXCL10), we observed no statistically significant differences between WT and Polg^D257A^ mice (Fig. S2G). These data argue that at day 21 post-Mtb infection, the Polg^D257A^ mutation drives immune changes in the lung without making a significant impact on the circulating immune milieu.

### Necrotic debris dominates Polg^D257A^ mutator mouse lungs during Mtb infection

**Fig 4:**
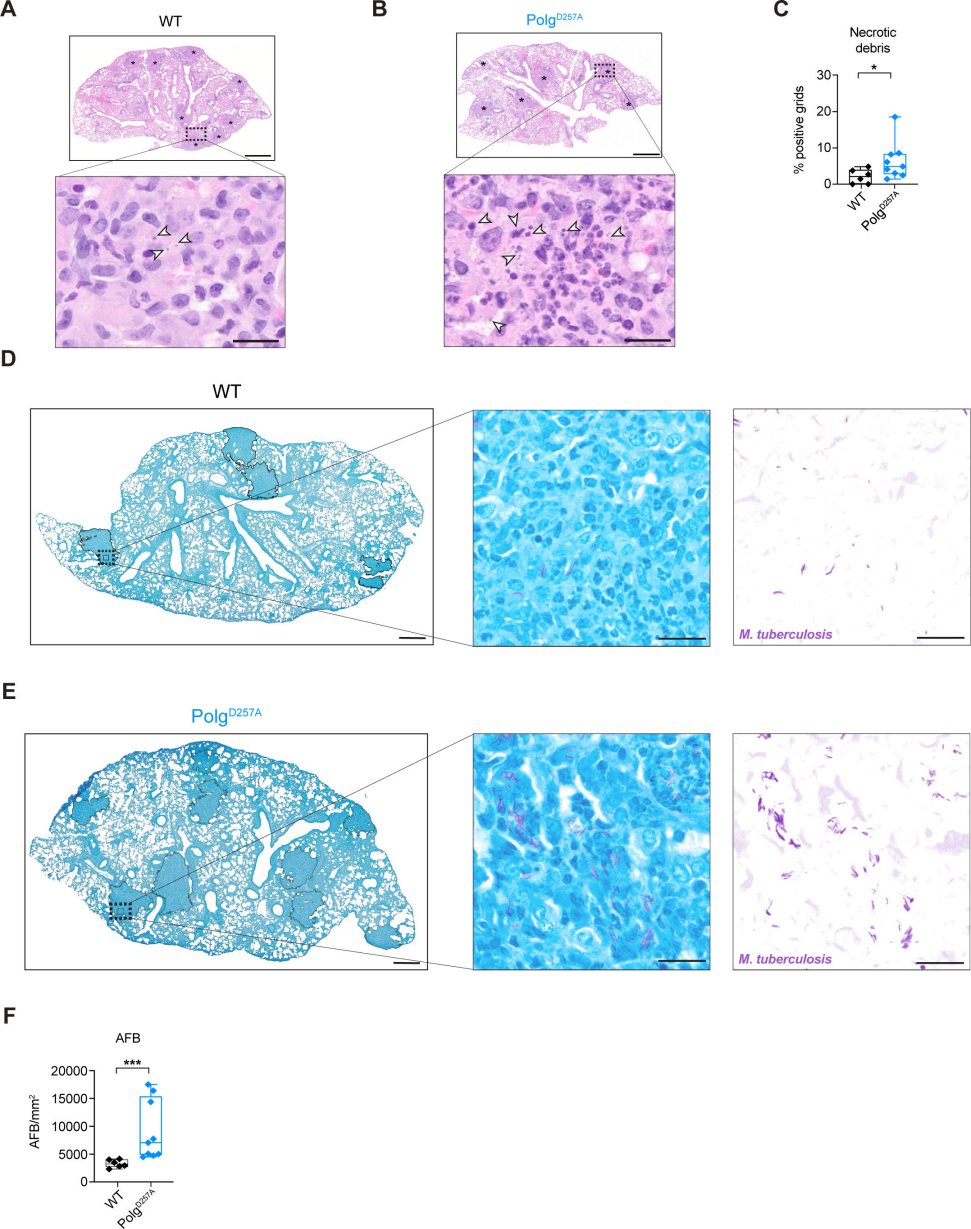
Polg^D257A^ mutator mice exhibit increased necrotic debris and higher bacterial burden in areas of necrosis during Mtb infection. Hematoxylin and eosin staining of WT (**A**) and Polg^D257A^ (B) mice lungs at day 21 post-Mtb infection. Asterisks denote areas consolidated and infiltrated by inflammatory cells. White arrows denote necrotic debris (extracellular, circular, dense structures of dead cells). Upper panel scale bar is 800 µm. Lower panel scale bar is 10 µm. (**C**) Quantification of necrotic debris using H&E staining of WT and Polg^D257A^ mice lungs at day 21 post-Mtb infection. Each data point represents the percentage of positive grids with necrotic debris in WT and Polg^D257A^ mice Mtb-infected lungs. Acid-fast Bacilli (AFB) staining of WT (**D**) and Polg^D257A^ (E) mice lungs at day 21 post-Mtb infection. Black outlines denote consolidated areas of inflammatory infiltrates in the left panel. The middle and right panels represent insets of AFB staining and color deconvolution. Left panel scale bar is 800 µm. Middle and right panels scale bar is 20 µm. (**F**) Quantification of AFB bacilli in black outlined consolidated areas of inflammatory infiltrates (see “Quantification of acid-fast bacteria staining” in Materials and Methods). Statistical analysis: *P < 0.05, **P < 0.01, ***P < 0.001, and ****P < 0.0001. Statistical significance was determined for panels **C and F** using Mann-Whitney *U* test.

Having observed altered immune cell infiltration and inflammatory transcript abundance in Mtb-infected Polg^D257A^ mutator mice, we next looked more carefully at immunopathology in the lungs. We and others have previously associated necrotic debris, characterized by neutrophil clusters with granular nuclear debris from dead cells, with poor tuberculosis outcomes in mice (26, 40). Accordingly, we observed increased clusters of necrotic debris in Mtb-infected Polg^D257A^ mutator mice lungs compared to WT (Fig. 4A through C, white arrows). Because necrosis is associated with exuberant Mtb growth and virulence (22, 40), we next used acid-fast staining to look for Mtb bacilli in areas of consolidated inflammatory infiltrates **(**Fig. 4D and E, black outline). We denoted instances of growth characteristic of cording, a virulence-related form of Mtb growth associated with necrosis, specifically in the lungs of Polg^D257A^ mutator mice (Fig. 4D and E) (19, 40). To quantify the number of Mtb bacilli in areas of neutrophil clusters and necrotic debris, we developed an image analysis workflow to quantify the number of acid-fast positive bacilli (AFB) within selected regions of interest in lung sections. Using this approach, we measured significantly more Mtb bacilli in necrotic regions of Polg^D257A^ mutator mice lungs compared to WT (Fig. 4F). These findings suggest that areas of necrotic debris that dominate Polg^D257A^ mutator mice lungs correlate with a failure to restrict Mtb growth.

### Polg^D257A^ macrophages are prone to necrotic death and cell death in response to Mtb infection

**Fig 5:**
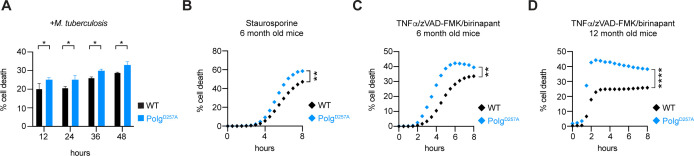
Polg^D257A^ mutator macrophages are prone to necrotic cell death. (**A**) Cell death in 6-month-old WT and Polg^D257A^ macrophages infected with Mtb for 48 h (MOI = 5). (**B**) Cell death in 6-month-old WT and Polg^D257A^ macrophages over a time course of apoptosis induction with 100 nM staurosporine. Cell death in 6-month-old (**C**) or 12-month-old (**D**) WT and Polg^D257A^ macrophages treated with 100 ng/mL TNFα, 500 nM Birinapant (SMAC mimetic), and 20 µM zVAD-FMK (pan-caspase inhibitor) to induce necroptosis. Cell death was measured by propidium iodide uptake (% cell death = propidium iodide positive/total cell count × 100%). Statistical analysis: *n* = 3 *P < 0.05, **P < 0.01, ***P < 0.001, and ****P < 0.0001. Statistical significance was determined for panel **A** using one-way ANOVA with Sidak’s post-test and panels **B–D** using two-way ANOVA with Tukey’s post-test.

To better understand the molecular mechanisms underlying the susceptibility of Polg^D257A^ mutator mice, we asked if their propensity to accumulate necrotic debris was observable only in the infected lung or if there was a cell-intrinsic propensity for Polg^D257A^ macrophages to undergo necrosis. To test this, we isolated bone marrow and differentiated macrophages from WT and Polg^D257A^ mutator mice at either 6 or 12 months of age. As mutations sustained by the Polg^D257A^ mutator mice are cumulative, these ages represent the typical “onset” and “late” stages of mitochondrial disease as they relate to human patients (27, 41). We first infected WT and Polg^D257A^ BMDMs from 6-month-old mice with Mtb (MOI = 5) and tracked cell death kinetics using propidium iodide (PI). After 24 h of infection, Polg^D257A^ macrophages were more prone to Mtb-induced cell death than WT controls (Fig. 5A). Because Mtb infection triggers several cell death pathways in macrophages (apoptosis [42], ferroptosis [24], and pyroptosis [20, 21]) and PI cannot distinguish between these forms of cell death, we next exposed cells to canonical cell death triggers to gain additional insight into the type of cell death that is enhanced in Polg^D257A^ BMDMs. To begin, we treated WT and Polg^D257A^ BMDMs from 6-month-old mice with the apoptosis inducer staurosporine, a protein kinase inhibitor that triggers apoptosis via aberrant caspase activation. In response to a low dose of staurosporine (100 nM), we observed slightly more propidium iodide incorporation in Polg^D257A^ BMDMs (Fig. 5B). These findings are consistent with previous work that found increased apoptotic markers associated with accelerated aging in Polg^D257A^ mutator mice (24). Next, we treated WT and Polg^D257A^ BMDMs with recombinant murine TNFα in combination with a SMAC mimetic (birinapant) and pan-caspase inhibitor to initiate RIPK1/RIPK3/MLKL-dependent necroptosis (43). Polg^D257A^ macrophages isolated from 6-month-old mice were highly susceptible to necroptosis (Fig. 5C), and this phenotype was enhanced in Polg^D257A^ BMDMs from 12-month-old mice (Fig. 5D). These findings suggest that Polg^D257A^ macrophages are more sensitized to necrotic forms of cell death, which may account for enhanced necrosis and Mtb dissemination *in vivo*.

## DISCUSSION

Because mitochondrial-associated mutations can increase the risk of life-threatening infections, there is a critical need to understand how altered mitochondrial homeostasis affects the immune response to pathogens. Here, we describe how a mutation in mitochondrial Polg, which results in the accumulation of mtDNA mutations and mtDNA instability, confers susceptibility to Mtb infection by enhancing necrosis. We demonstrate that in response to Mtb, Polg^D257A^ mutator mice fail to mount protective immune responses in the lung. We propose that this stems in large part from the propensity of Polg^D257A^ macrophages to preferentially undergo necrosis over other forms of programmed cell death when infected with Mtb. Enhanced necrosis is a likely driver for the pro-bacterial cellular immune milieu (high neutrophils and low M1 macrophages) we observe in Polg^D257A^ mutator mice. It may also directly support high levels of Mtb growth in Polg^D257A^ lungs. Studies from the Ramakrishnan lab (44, 45) have repeatedly linked necrosis to extracellular mycobacterial cording and poor disease outcomes in the zebrafish model. In human macrophages, Mtb replication is enhanced in cells undergoing late stages of necrosis that exhibit evidence of plasma membrane damage (46). Mtb has also been shown to thrive in macrophages that have phagocytosed Mtb-infected polymorphonuclear neutrophils (PMNs) that died via necrosis (40). Although we are unable to decisively assess whether Mtb bacilli in Polg^D257A^ lungs are extracellular due to limitations of H&E and AFB staining, our data convincingly correlate enhanced necrosis in Polg^D257A^ mutator mice with high Mtb bacterial burdens.

Over the course of these studies, we were surprised to find that despite harboring more bacteria in the lungs, spleen, and liver (Fig. 1), Polg^D257A^ mutator mice expressed lower levels of cytokines/chemokines overall (Fig. 3; Fig. S2). It is possible that the accumulation of mtDNA mutations and chronic stimulation of pattern recognition receptors by mitochondrial DAMPs render Polg^D257A^ immune cells refractory to immune signaling upon pathogen sensing (47). We previously reported a phenotype like this in LRRK2 KO macrophages, which have high basal type I IFN expression and are unable to induce ISG expression in response Mtb (48). It is also possible that the failure of Polg^D257A^ mutator mice to upregulate pro-inflammatory cytokines stems from rewiring of cellular metabolism and mitochondrial OXPHOS, which is uniquely regulated in Mtb-infected macrophages (49, 50). Previous work has linked the Polg^D257A^ mutation to altered type I IFN signaling in response to bacterial pathogens. Specifically, the West lab (30) reported that following 6–48 h post-intratracheal instillation of *Pseudomonas aeruginosa* strain O1 (PAO1), Polg^D257A^ mutator mice exhibited elevated levels of ISGs in the lung and BAL fluid. It is possible that upon initial encounter with Mtb, Polg^D257A^ mutator mice similarly hyperinduce ISGs and pro-inflammatory cytokines and that we are reporting on a different, chronic response at 21 days post-infection. However, it is also possible that the Polg^D257A^ mutation responds to and elicits an immune milieu to different pathogens in different ways. Indeed, we know that *Pseudomonas* and Mtb initially elicit type I IFN expression through distinct signaling pathways (TLR4/TRIF [51] and cGAS [52], respectively) and likely interface with mitochondria in distinct ways as well. Investigations of Mtb-infected Polg^D257A^ mutator mice at additional early time points may help us distinguish between these two potential explanations.

These studies were motivated, in part, by several genome-wide association studies (GWAS) that identified SNPs in mitochondrial genes that confer susceptibility to mycobacterial infection in humans. Most of these studies looked at patients with leprosy, which is caused by *Mycobacterium leprae*, a close cousin of Mtb. Mutations in genes like *PARK2*, which encodes the mitophagy factor Parkin (53, 54), *TFAM*, which encodes a histone-like protein of the mitochondrial genome (11), as well as *POLG* (11), have been linked to leprosy susceptibility or severity of disease. The Mtb phenotype we report is consistent with GWAS, which identified a human SNP in *POLG* associated with multibacillary leprosy—a form of the disease characterized by high bacterial loads in lesions (11). Collectively, our study bolsters confidence in these GWAS and provides new insight into the potential mitochondria-associated mechanisms driving altered susceptibility to mycobacterial disease.

### Limitations of the study

One drawback of the Polg^D257A^ mutator mouse model is that the mice accumulate mutations throughout the entire mtDNA genome over time (55). Thus, one cannot ascribe pathological phenotypes to a specific defect in a single mitochondrial gene. Likewise, although human patients with mutations in the exonuclease “proofreading” domain of *POLG* have been linked to diseases such as Alpers and Leigh syndrome (56, 57), Polg^D257A^ mutator mice do not mirror a specific model of mitochondrial disease. Given these caveats, future investigations may wish to probe Mtb pathogenesis in mice that more accurately model mitochondrial mutations in humans. Likewise, it will be interesting to determine the susceptibility of the Polg^D257A^ mutator mice at later time points during Mtb infection since mtDNA mutations accumulate with age in these mice. Our bacilli staining in particular hints at a bifurcation in our mouse data, whereby certain mice harbor incredibly high Mtb burdens in necrotic lesions (Fig. 4). It is tempting to speculate that these are mice that have accumulated the most mtDNA mutations—a hypothesis that can be tested by sequencing mtDNA from the lungs of the Polg^D257A^ mutator mice. Despite these limitations, our study adds to a growing literature arguing that mitochondrial health is required to generate protective immune responses to Mtb. Accordingly, we propose that disruption to mitochondrial homeostasis, via mutation, metabolic disease, stress, etc., plays a critical role in dictating the spectrum of tuberculosis disease in humans.

## MATERIALS AND METHODS

### Mouse husbandry and strains

*Polg*^*D257A/D257A*^ C57BL/6J mutator mice were purchased from the Jackson laboratory. *Polg*^*D257A/+*^ breeder pairs used to generate *Polg*^*+/+*^ and *Polg*^*D257A/D257A*^ experimental mice were obtained from male *Polg*^*D257A/+*^ to female C57BL/6J crosses. All mice used in experiments were compared to age- and sex-matched controls and fed 4% standard chow. Littermate controls were used in all experiments. For *ex vivo* BMDM experiments, male mice 6 and 12 months of age were used. For *in vivo* Mtb infections, mice were infected at 12 weeks of age.

### M. tuberculosis

The Erdman strain was used for all *M. tuberculosis* infections. Low-passage lab stocks were thawed for each experiment to ensure that virulence was preserved. *M. tuberculosis* was cultured in roller bottles at 37°C in Middlebrook 7H9 broth (BD Biosciences) supplemented with 10% OADC (BD Biosciences), 0.5% glycerol (Fisher), and 0.1% Tween-80 (Fisher) or on 7H10 plates. All work with *M. tuberculosis* was performed under Biosafety level 3 containment using procedures approved by the Texas A&M University Institutional Biosafety Committee.

### Primary cell culture

Bone marrow-derived macrophages were isolated by flushing mouse femurs and tibias with 10 mL DMEM and 1 mM sodium pyruvate. Cell suspensions were centrifuged at 400 rcf for 5 min and resuspended in BMDM media (DMEM, 20% FBS [MilliporeSigma], 1 mM sodium pyruvate [Lonza], and 10% MCSF conditioned media [Watson lab]). Cells were plated on non-TC-treated plates and differentiated at 37°C and 5% CO_2_. On day 3 following plating, cells were fed 50% additional volume with BMDM media. Cells were harvested at day 7 with 1× PBS EDTA (Lonza).

### *M. tuberculosis* infection

For *M. tuberculosis* infections *ex vivo*, low passage Mtb was prepared by growing it to log phase (OD_600_ 0.6–0.8). Bacterial cultures were spun at 58 rcf for 5 min to remove large clumps. The bacteria were then pelleted at 2,103 rcf for 5 min and washed with 1× PBS. The wash step was repeated twice. The resuspended bacterial cultures were sonicated at 70% amplitude for 10 s and repeated three times (Branson Ultrasonics Corp.) followed by a low-speed spin (58 rcf) to remove remaining clumps. The bacteria were diluted in DMEM (Hyclone) + 10% horse serum (Gibco) for *ex vivo* infections or 1× PBS for *in vivo* infections. For *ex vivo* infections, plates containing cells and bacteria were spun at 234 rcf for 10 min to synchronize infection. Fresh BMDM media was then added to the cells.

### Plate-based assays for cell death

BMDMs were plated in 96-well clear bottom plates (Corning) at 2.5 × 10^4^ cells/well in 50 µL of media. Following cell adherence (1 h), an additional 25 µL was added to each well. The following day, media were removed, and respective agonists were used to initiate various cell death modalities. For cell death assays, 5 µg/mL PI (ThermoFisher) was added at the time of adding cell death agonists. Total cell numbers used for normalization were counted on a subset of cells using NucBlue (ThermoFisher) in 1× PBS. Live cell imaging was done using 4× magnification on either a Lionheart plate reader or Cytation 5 (Biotek). Post-run analysis was conducted using Gen5 version 3.15 (Biotek) software. For necroptosis induction, cells were activated with 100 ng/mL recombinant murine TNFα (PeproTech), 500 nM SMAC mimetic birinapant (Selleck Chemicals), and 20 µM pan-caspase inhibitor z-VAD-FMK (R&D Systems). Apoptosis was induced with 100 nM stauroporine (Tocris Bioscience).

### *In vivo* Mtb infections

All infections were performed using procedures approved by Texas A&M University Institutional Care and Use Committee. The Mtb inoculum was prepared as described above. Age- and sex-matched mice were infected via inhalation exposure using a Madison chamber calibrated to introduce 100–200 CFUs per mouse. For each infection, approximately three mice were euthanized immediately, and their lungs were homogenized and plated to verify an accurate inoculum. Infected mice were housed under BSL3 containment and monitored daily by lab members and veterinary staff. At the indicated time points, mice were euthanized, and tissue samples were collected. Blood was collected in serum collection tubes, allowed to clot for 1–2 h at RT, and spun at 1,000 rpm for 10 min to separate serum. Organs were divided to maximize infection readouts; CFUs: left lobe lung and 1/2 spleen; histology: two right lung lobes and 1/4 spleen; RNA: post-caval lobe and 1/4 spleen; flow: right inferior lobe. For histological analysis, organs were fixed for 24 h in 10% neutral-buffered formalin and moved to ethanol (lung and spleen). Organs were further processed as described below. For cytokine transcript analysis, organs were homogenized in Trizol, and RNA was isolated as described below. For CFU enumeration, organs were homogenized in 5 mL PBS + 0.1% Tween-80, and serial dilutions were plated on 7H10 plates. Colonies were counted after plates were incubated at 37°C for 3 weeks.

### Multiplex immunoassay

The levels of a panel of cytokines and chemokines in serum were measured using a 12-plex ProcartaPlex Multiplex Immunoassay according to the manufacturer’s instructions (Invitrogen, Carlsbad, CA, USA). Standards were prepared to determine the concentration of cytokines and chemokines in the samples. The samples were run on a Magpix instrument and analyzed with Milliplex Analyst version 5.1 software. For data analysis, a five-parameter logistic curve fitting method was applied to the standards, and the sample concentrations were extrapolated from the standard curve. All experiments using infectious agents were conducted in BSL-3 or ABSL-3 facilities of the University of Tennessee Health Science Center Regional Biocontainment Laboratory (UTHSC RBL) compliant with protocols that were reviewed and approved by the UTHSC Institutional Biosafety Committee (IBC) and the Institutional Animal Care and Use Committee (IACUC).

### Gene expression analysis by RT-qPCR

For lung tissue, RNA was isolated using Direct-zol RNAeasy kits (Zymogen). cDNA was synthesized with Bio-Rad iScript Direct Synthesis kits (BioRad) per the manufacturer’s protocol. RT-qPCR was performed in triplicate wells using PowerUp SYBR Green Master Mix. Data were analyzed on a QuantStudio 6 Real-Time PCR System (Applied Biosystems), and quantification of gene expression was performed using a standard curve and normalized to *Actb* expression levels. qRT-PCR primer sequences can be found in Table 1.

**TABLE 1:** qRT-PCR primer sequences

Gene	FWD primer	REV primer
Tnf	ATGGCCTCCCTCTCATCAGT	GTTTGCTACGACGTGGGCTA
Nos2	GCAGCACTTGGATCAGGAA	GAAACTTCGGAAGGGAGCAA
Ifng	CTCTTCCTCATGGCTGTTTCT	CGCTTATGTTGCTGATGG
Nlrp3	AAAATGCCTTGGGAGATCCA	AAGTAAGGCCAGAATTCACC
Cxcl9	TGTGGAGTTCGAGGAACCCT	TGCCTTGGCTGGTGCTG
Cxcl10	CCAAGTGCTGCCGTCATTTTC	GGCTCGCAGGGATGATTTCAA
Il1b	GGTGTGTGACGTTCCCATTA	ATTGAGGTGGAGAGCTTTCAG
Il6	CCAGAGTCCTTCAGAGAGATACA	CCTTCTGTGACTCCAGCTTATC
Il1ra	CAGCTCATTGCTGGGTACTT	CTCAGAGCGGATGAAGGTAAAG
Isg15	GAGCTAGAGCCTGCAGCAAT	TTCTGGGCAATCTGCTTCTT
Rsad2	ATAGTGAGCAATGGCAGCCT	AACCTGCTCATCGAAGCTGT
Cxcl1	CCGAAGTCATAGCCACACTCA	CTCCGTTACTTGGGGACACC
Cxcl5	TGCCCTACGGTGGAAGTCAT	AGCTTTCTTTTTGTCACTGCCC
Ifnb1	TCCGAGCAGAGATCTTCAGGAA	TGCAACCACCACTCATTCTGA
beta-Actin	GGTGTGATGGTGGGAATGG	GCCCTCGTCACCCACATAGGA

### Flow cytometry

For *ex vivo* analysis of lung cell populations at 21 days post-Mtb infection (WT [*n* = 6] and Polg^D257A^ [*n* = 8]), the right inferior lobe was isolated and washed twice in 1× PBS. The lung was then minced and digested in digestion buffer (70 µg/mL Liberase [Roche] and 50 µg/mL DNase I [Worthington Biochemical] in RPMI 1640 [HyClone]) for 30 min at 37°C and 5% CO_2_. Single suspensions were achieved by consecutively filtering homogenates through 70 and 40 µm cell strainers. Live/dead staining was performed using Ghost dye 510 (Tonbo). Fc receptors were blocked using CD16/CD32 monoclonal antibody (eBiosciences). Cells were stained with antibodies against surface proteins CD11b (BV421, BDBiosciences), CD11c (BV605, BioLegend), CD45 (BV785, BioLegend), CD170 (eFluor-488, eBiosciences), CD43 (PE, BD Biosciences), Ly6G (PerCP-Cy5.5, eBiosciences), Ly6C (APC, eBiosciences), CD206 (APCeFLuor-700 eBiosciences), and CD86 (APCeFluor-780, eBiosciences). Cells were washed two times before fixing in 4% PFA for 15 min at RT. Following fixation, cells were washed twice with 1× PBS and incubated overnight at 4°C. Total lung cell counts were based on live single cells (Ghost low/−) in 200 mL or 1/3 lung lobe. Forward scatter (FSC)/ Side scatter (SSC) was used to differentiate macrophage (CD45^+^ CD11b^+^ Ly6G^−^ CD11c^low/−^ SSC^hi^ FSC^mid^) and monocyte (CD45^+^ CD11b^+^ Ly6G^−^ CD11c^low/−^ SSC^lo^ FSC^mid^). Flow cytometry was performed on the LSR Fortessa X20, and FCS Express software was used for post-acquisition analysis. See Fig. S1A for flow gating strategy.

### Protein quantification by immunoblot

Cells were washed with 1× PBS and lysed in 1× RIPA buffer with protease and phosphatase inhibitors (Thermo Scientific), with the addition of 1 U/mL Benzonase (Millipore) to degrade genomic DNA. Proteins were separated by SDS-PAGE Any kD mini-PROTEAN TGX precast gels (Bio-Rad) and transferred to 0.45 µm nitrocellulose membranes (GE Healthcare). Membranes were blocked for 1 h at RT in TBS with 5% BSA. Blots were incubated overnight at 4°C with the following antibodies: β-tubulin (Abcam, ab179513) and VIPERIN (Millipore, MABF106). Membranes were washed and incubated with appropriate secondary antibodies for 2 h at RT prior to imaging on a LiCOR Odyssey Fc Dual-Mode Imaging System. For protein isolated from tissue, lung homogenates were lysed in 1% SDS lysis buffer and then boiled for 30 min at 95°C. Protein per sample was quantified using a bicinchoninic acid assay (Thermo Fisher Scientific, PI23235). Thirty micrograms of total protein was loaded into 10% SDS-polyacrylamide gels and transferred onto 0.22 µm polyvinylidene difluoride membranes, dried, and then incubated overnight at 4°C with the following primary antibodies in 1× PBS containing 1% casein: VIPERIN (Abcam, ab107359), IL-1β (Cell Signaling Technology, 12242), NLRP3 (Cell Signaling Technology, 15101), GAPDH (ProteinTech, 60004-1-Ig), and Actin (ProteinTech, 66009-1-Ig). Next, membranes were washed and incubated with horseradish peroxidase (HRP)-conjugated secondary antibodies for 1 h at room temperature. Luminata Crescendo Western HRP Substrate (Millipore, WBWR0500) was used to visualize proteins.

### Histopathology

Mtb-infected mouse lungs were fixed with 10% neutral formalin and processed, embedded in paraffin, and cut into 5 µm sections and stained with H&E or AFB (AML Laboratories). A boarded veterinary pathologist performed a blinded evaluation of lung sections for inflammation. To quantify the percentage of lung fields occupied by histiocytic inflammatory infiltrates, scanned images of a lobe of each lung were analyzed using QuPath Bioimage analysis version 0.4.3 to determine the total cross-sectional area of inflammatory foci per total lung cross-sectional area. Additional criteria were evaluated by dividing the digital images into 500 × 500 µm grids and counting the percentage of squares containing neutrophils and neutrophils arranged in clusters (>5 neutrophils in close approximation). Necrotic debris represents the nuclear remnants of dead cells. Necrotic debris was classified as extracellular, circular, densely basophilic structures that were variably sized, though always smaller than an erythrocyte (less than 6 µm), and frequently seen in association with neutrophil clusters. WT and Polg^D257A^ mice lung sections were divided into 500 × 500 µm grids and evaluated by counting the percentage of squares based on the presence or absence of necrotic debris.

### Quantification of acid-fast bacteria staining

To directly quantify the bacillary burden within regions of inflammatory infiltrate, a board certified veterinary pathologist used QuPath (version 0.4.3) to manually annotate the total area of inflammatory regions in each acid-fast stained lung section. Subsequently, a script was used to randomly sample ten 100 × 100 µm square regions of interest within these areas. In these regions, a reviewer blinded to the study details manually counted the carbolfuchsin-positive bacilli.

### Statistical analysis

All data are representative of two or more independent experiments with *n* = 3 or greater unless specifically noted in the figure legends. For all quantifications, *n* represents the number of biological replicates, either the number of wells containing cells or the number of mice. Error bars represent SEM. For *in vitro* assays, statistical significance was determined using either a two-tailed Student’s unpaired *t* test or two-way ANOVA with Tukey’s *post hoc* test. For *in vivo* mouse infections, significance was determined using a Mann-Whitney *U* test based on the assumption that samples (mice) followed a non-normal distribution. The specific statistical test used to determine the significance for each experiment is listed at the end of the figure legends. All calculations of significance were determined using GraphPad Prism (version 10) Software expressed as *P* values. The threshold for significance was determined by a *P* value of < 0.05, and annotated as *P < 0.05, **P < 0.01, ***P < 0.001, and ****P < 0.0001. For *in vivo* infection mouse experiments, we estimated that detecting a significant effect requires two samples to differ in CFUs by 0.7e^10^. Using a standard deviation of 0.3e^10^ for each population, we calculated that a minimum size of five age- and sex-matched mice per group per time point is necessary to detect a statistically significant difference by a *t*-test with alpha (two-sided) set at 0.05 and a power of 80%. Therefore, we used a minimum of five mice per genotype per time point to assess infection-related readouts.

## SUPPLEMENTAL MATERIAL

Supplemental material iai.00324-24-s0001.pdf Fig. S1 and S2.

ASM does not own the copyrights to Supplemental Material that may be linked to, or accessed through, an article. The authors have granted ASM a non-exclusive, world-wide license to publish the Supplemental Material files. Please contact the corresponding author directly for reuse.
